# A Shared Intentionality Account of Uniquely Human Social Bonding

**DOI:** 10.1177/17456916231201795

**Published:** 2023-10-26

**Authors:** Wouter Wolf, Michael Tomasello

**Affiliations:** 1 Department of Developmental Psychology, Utrecht University; 2 Department of Psychology and Neuroscience, Duke University; 3 Department of Developmental and Comparative Psychology, Max Planck Institute for Evolutionary Anthropology, Leipzig, Germany

**Keywords:** evolutionary psychology, interpersonal relations, other, shared intentionality, social bonding, social cognition

## Abstract

Many mechanisms of social bonding are common to all primates, but humans seemingly have developed some that are unique to the species. These involve various kinds of interactive experiences—from taking a walk together to having a conversation—whose common feature is the triadic sharing of experience. Current theories of social bonding have no explanation for why humans should have these unique bonding mechanisms. Here we propose a shared intentionality account of uniquely human social bonding. Humans evolved to participate with others in unique forms of cooperative and communicative activities that both depend on and create shared experience. Sharing experience in these activities causes partners to feel closer because it allows them to assess their partner’s cooperative competence and motivation toward them and because the shared representations created during such interactions make subsequent cooperative interactions easier and more effective.

Mammals evolved to live in social groups. But group life often means social competition for food and other resources. In this context, it pays to have friends, and indeed, long-term research on various primate species has established that one of the most important factors in the health, longevity, and reproductive success of both male and female primates is the positive social bonds they create with others (Silk, 2007; Silk et al., 2003; Snyder-Mackler et al., 2020). The human primate is no exception, given that humans develop strong needs for social connection that, if unfulfilled, severely affect their physical and mental well-being (Baumeister & Leary, 1995; Bowlby, 1969; Williams, 2007).

Human social networks, however, seem to differ from those of other primates in important ways. Most obviously, human social networks are relatively large compared with those of other primates, suggesting that they are maintained by uniquely efficient social-bonding activities (Dunbar, 1993, 2020; Shultz & Dunbar, 2007). But the precise psychological nature of humans’ unique social-bonding activities and how these came to be evolutionarily are open questions for which current psychological theories of human social bonding provide no answer. Here we propose that the evolution of humans’ unique forms of social bonding is part and parcel of the evolution of their unique forms of cooperative social interaction facilitated by shared intentionality. Specifically, we propose that individuals bond with partners in cooperative activities not only by sharing mental states during these experiences but also by actively creating shared representations of their experiences as a unique and highly effective form of triadic social engagement.

## Current Psychological Theories of Social Bonding

A complete psychological theory of (the evolution of) human social bonding needs to account not only for similarities in social bonding in phylogenetically related species but also for the uniqueness of the psychology underlying human social bonding that explains humans’ relatively large social networks and peculiar social-bonding activities and how this psychology manifests in social networks and institutions across cultures in response to local ecological challenges. The two classic psychological theories of social bonding are attachment theory (Bowlby, 1969) and belongingness theory (Baumeister & Leary, 1995). Both are important and well-supported theories that provide deep insights into the nature of human social bonding. But neither explicitly addresses its species-unique aspects.

Attachment theory began with the observation that human infants and young children, like the juveniles of many creatures, seek proximity to their caregivers in situations of uncertainty and/or stress (Bowlby, 1969). How caregivers respond to these overtures shapes the bonds they create with them and serves as a working model for social relationships later in life (Kirkpatrick & Davis, 1994). Attachment theory is still one of the dominant developmental frameworks for understanding children’s social bonds with their caregivers and social bonding in later stages in life. It addresses a wide range of questions concerning the functions, emotional dynamics, and developmental pathways of human social bonding based on need responsiveness and stress reduction (Hazan & Shaver, 1994).

In addition, social psychologists have also long discussed humans’ fundamental need for belonging. In his hierarchy of needs, Maslow (1968) already speculated that humans have a fundamental need for love and belongingness. This idea rapidly gained traction after Baumeister and Leary’s (1995) seminal article on the need to belong, which made a convincing case that the need to belong, conceptualized as a need for frequent interaction and persistent caring, permeates most, if not all, of humans’ social behavior, cognition, and emotions and thus their physical and mental well-being (for a review, see Williams, 2007). Theories of belonging currently provide the most important framework for research on social bonding in adult humans and research on social motivation in general (e.g., see Deci & Ryan, 2012).

Both attachment theory and belongingness theory emphasize that human social-bonding systems are evolutionary adaptations, rooted in the need for social protection and for navigating social competition via coalitions and alliances (Baumeister & Leary, 1995; Bowlby, 1969). Because humans share both of these characteristics with many other mammals and primates (Dunbar, 2020), these theories propose a high degree of phylogenetic similarity between humans’ social-bonding systems and those of their evolutionary relatives. Comparative research has supported this idea, having found that nonhuman primates also form attachments to caregivers (Clay et al., 2015) and show a strong need to belong, which, if thwarted, causes stress and negatively affects well-being (Harlow, 1958; Harlow et al., 1965; Sapolsky, 2005). Moreover, humans seem to share much of the neuroendocrinological architecture related to attachment and belonging with other primates, including those related to oxytocin (Crockford et al., 2013) and beta-endorphins (Keverne et al., 1989).

Given that both humans and nonhuman primates create attachment and have a sense of belonging, it is no surprise that humans also share some social-bonding mechanisms and activities with them. These include seeking physical proximity (Ancillotto et al., 2015; Arseneau-Robar et al., 2018; Micheletta et al., 2012; Schamberg et al., 2016; Zhang et al., 2012), engaging in behavioral mimicry (Chartrand & Bargh, 1999; Chartrand & Lakin, 2013; Chartrand & van Baaren, 2009; Kühn et al., 2010; Paukner et al., 2009; although nonhuman primates seem to do so less frequently than humans; see Call & Carpenter, 2003, 2009; Paukner et al., 2009), physical touch (Arseneau-Robar et al., 2018; Behncke, 2015; Dunbar, 1991, 2010; Gallace & Spence, 2010, 2016; Hare et al., 2015; Jefferies et al., 2018; Lehmann et al., 2007; Wittig et al., 2008), and experiencing behavioral synchrony (Fellner et al., 2006; Hove & Risen, 2009; Howard et al., 2021; Mogan et al., 2017). Attachment and belongingness theories thus provide a compelling argument that in many ways, the psychology underlying human social bonding is similar to that of closely related species and should therefore be perceived (and studied) as part of a phylogenetic continuum.

## The Puzzle of Uniquely Human Social Bonding

But something about human social bonding does seem to be different. Over the last several decades, evolutionary anthropologists and psychologists have noted that human social networks are considerably larger than those of other primates (Dunbar, 2020; Hill & Dunbar, 2003; Kasper & Voelkl, 2009; Lehmann et al., 2007; Shultz & Dunbar, 2010). To maintain such large social networks, given the time constraints of all the other activities necessary to survive (Dunbar et al., 2009), humans needed to find novel, more efficient ways to create social closeness with other individuals (Dunbar, 2012; Dunbar et al., 2009).

And so they have: To bond with others, humans, for example, use linguistic communication. Research has shown that social closeness is effectively generated by exchanging personal information (Aron et al., 1997) or gossiping about others (Bosson et al., 2006; Dunbar, 2004). In addition, humans also engage in many unique social-bonding activities that are not centered around conversations, such as laughing together (Caruana, 2017; Dunbar, 2012), making music together (Pearce et al., 2015; Weinstein et al., 2016), playing team sports (Artinger et al., 2006) or (video) games (Dabbish, 2008; Depping & Mandryk, 2017; Zheng et al., 2002), dancing together in synchrony (Cirelli et al., 2017; Hove & Risen, 2009; Mogan et al., 2017; Tarr et al., 2015, 2016), participating in rituals (Charles et al., 2021; Singh et al., 2020), and even watching something together in joint attention with others (Rennung & Göritz, 2015; Wolf et al., 2015; Wolf & Tomasello, 2020b).

The nature of these idiosyncratic social-bonding activities suggests that (at least part of) the psychology underlying human social bonding is different than in other animals. Thus, although attachment and belongingness theories provide a compelling account for why human social bonding is so similar to that of phylogenetic-related species in so many ways, these theories do not provide a psychological explanation for the unique psychology underlying human social bonding that enables people to live in such large social networks and to engage in uniquely effective social-bonding activities. Furthermore, these theories also do not provide an evolutionary explanation for how and why this unique social-bonding psychology emerged in human evolutionary history in the first place.

## A Shared Intentionality Account of Uniquely Human Social Bonding

To explain uniquely human social bonding, it must be seen as part of a much broader pattern of uniquely human social cognition and behavior. One proposal is that humans’ uniquely complex forms of communication, collaboration, and cultural learning are all manifestations of an adaptation for a uniquely cooperative psychology of “shared intentionality” (Angus & Newton, 2015; Call, 2009; Tomasello, 2010, 2014, 2019; Tomasello & Carpenter, 2007; Tomasello & Rakoczy, 2003; Tomasello & Vaish, 2013).

The core idea is that during a crucial part of their evolutionary history, humans had to adapt to a rapidly changing ecology (Tomasello et al., 2012). Around the time when the human genus first emerged, human ecology saw a shrinkage in the prevalence of closed forests, which changed the human environment into a more grassland-based ecology (Bobe et al., 2002), which, in turn, also caused a radiation of terrestrial monkeys (Elton, 2007). This relatively sudden ecological change combined with increased competition with other species made it harder for humans to obtain resources that were previously easily acquired individually. This forced humans in an evolutionary niche in which a substantial part of resource acquisition became dependent on sources that could be acquired only through collaboration. The emergence of this evolutionary niche created a level of interdependence (Tomasello et al., 2012) that requires high levels of coordination and collective decision-making. In addition, to keep individuals motivated to invest in such costly forms of cooperation, spoils must be shared efficiently in a way that minimizes the risk for aggression and conflict while potential free riders are kept in check through punishment or ostracism.

As a consequence, humans were socially selected for their capacity to develop and learn social-cognitive skills that facilitated communication, collaboration, and social learning. Specifically, they developed the capacity and motivation to share intentional states (i.e., mental states directed at something or someone, e.g., attention, intentions, directed emotions, or beliefs; see Brentano, 1874) with others. This capacity allows individuals to merge their converging mental states into a single corepresentation (i.e., “We experience X”), making it easier to predict a partner’s future behavior (Shteynberg et al., 2023).

Crucially, humans also seem to be very capable and motivated to create a mutualistic awareness of these intentional states being shared (Tomasello & Carpenter, 2007). This mutual awareness creates a shared or collective representation (e.g., “We are aware that we attend to X”; cf. Grice, 1957), causing the partnership to become a joint agent in which both individuals are aware they perceive, intent, feel, and believe things together (Shteynberg et al., 2023; Siposova & Carpenter, 2019). To create such shared metacognitive representations (e.g., “I know we both know that we feel X”), both individuals have to do more than simply observe the other individual reacting to X. They also need to express the psychological impact this experience made on them to others and see their partner acknowledge this expression so that they know their partner knows they know how they feel (e.g., through communicative eye contact; Siposova & Carpenter, 2019).

This joint agent undergirded by shared representations creates mutualistic expectations: “We both know we both want X, therefore, we expect both of us to behave in accordance with achieving X, for example, by doing Z.” Moreover, it removes some plausible deniability for people wishing to defect: “If I know you know I want X, arguing I did not do Z because I did not want X is no longer a plausible excuse for noncooperative behavior, and if I still decide to do so, this might have negative consequences for my reputation as a reliable partner” (in line with Tomasello, 2020). Thus, creating shared representations facilitates collective decision-making and makes defectors more identifiable, facilitating more effective punishment.

### Ontogeny

Crucially, these capacities and motivations are not slowly acquired throughout childhood and adolescence but, instead, emerge very early in ontogeny, some already in infancy (Higgins, 2016; Tomasello, 2019; Tomasello & Carpenter, 2007). From around 1 year of age, children do not merely follow the gaze of others but are actively attempting to share their attention with others (Bakeman & Adamson, 1984; Liszkowski et al., 2004). Even prelinguistic children already do so by using cooperative communication such as pointing or using other types of gestures. When producing such communicative acts, even 1-year-olds already take into account their partner’s knowledge and attitudes (Liszkowski et al., 2007a, 2007b, 2008). In addition, 14-month-olds already interpret the meaning of communicative acts based on the experiences they have shared with the communicator in a special way, that is, only when both them and the communicator were aware of this experience being shared (Moll et al., 2007, 2008).

These social-communicative skills then facilitate the creation of joint attention, reference, and goals to be used in joint commitments at around 18 months of age (and under certain circumstances, already at 14 months; Warneken et al., 2006; Warneken & Tomasello, 2007), which is even more clearly illustrated by the tendency of slightly older children (2.5 years old) to persist in achieving a collaborative shared goal even after their individual goal had been achieved (Hamann et al., 2012). Research with slightly older children (5–7) also showed that young children seem to respond specifically cooperative when a partner attempts to communicate mutual awareness of their shared goals through communicative eye contact (Siposova et al., 2018). Finally, these social-communicative capacities also manifest itself in children’s sensitivity to pedagogy and instructed learning (Tomasello & Carpenter, 2007). Fourteen-month-olds are already more prone to imitate specific actions when these actions are specifically demonstrated for them, whereas without instructions, they more often attempt to copy the result (Csibra & Gergely, 2006), suggesting that they see instructional learning as a collaborative social activity.

### Phylogeny

The social-cognitive skills allowing people to engage in shared intentionality do not only emerge early in ontogeny but also seem, at least in part, unique to humans. For example, although great apes, as well as many other animals, seem to know what others can and cannot see and are quite competent at following others’ gaze (Bräuer et al., 2007; Hare et al., 2000; Karg et al., 2015), they do not seem to use mutualistic shared representations to infer a referent of others’ gaze more precisely (Tomasello & Carpenter, 2005). In addition, great apes seem not to take into account shared representations when interpreting gestures in a hiding-and-finding game (Call & Tomasello, 2005). Moreover, research on helping during collaborative and noncollaborative interactions has shown that great apes do not help partners with whom they are collaborating more often than individuals with whom they are not collaborating (Greenberg et al., 2010), suggesting that great-ape collaboration does not seem to rely on a mutually manifest shared plan or goal during collaboration. Finally, great apes also do not seem to engage in instructional teaching and learning among themselves (Tomasello & Herrmann, 2010).

### Shared intentionality and social bonding

It thus seems like people’s evolutionary history has caused the species to develop a unique set of social-cognitive skills that has transformed the way people communicate, cooperate, and learn from others. Here, we argue that these social-cognitive skills have also fundamentally changed the way in which people bond with other individuals. One crucial feature most, if not all, uniquely human social-bonding activities have in common is that they are centered around shared experiences. In fact, in many human social-bonding activities, an external stimulus often seems deliberately created solely for the purpose of sharing the experience of that stimulus with partners (e.g., music for dancing). For people to share their experience during such social-bonding activities, they need to engage in this activity and infer that the psychological impact this experience makes on others is similar to the psychological impact it made on them. That is, people feel more connected when they perceive the same music, want to respond in the same way (e.g., dance), experience similar emotions while doing it, and form similar beliefs (e.g., this music is well suited for dancing) as a consequence.

Several studies have supported this idea: Experiments in which participants sitting in close proximity either attend to something together or attend to something individually have shown that sharing the experience of jointly attending to something together causes individuals to feel closer to one another (Haj-Mohamadi et al., 2018; Rennung & Göritz, 2015; Wolf et al., 2015). This even seems to be the case for children as young as 2.5 years old: Children who watched a video together with an adult experimenter were more willing to interact with that experimenter afterward (i.e., approached the experimenter faster) than children who watched the video individually while the experimenter sat next to them reading a book (Wolf & Tomasello, 2020b).

But is social bonding through sharing experiences truly unique to humans? Do other animals not create a social connection by sharing experiences at all? Recent research suggests that it is not that simple. Studies in which the experimental paradigms of research with young children were adapted to be suitable for great apes showed surprisingly similar results (Wolf & Tomasello, 2019): When chimpanzees and bonobos watched a video together with a novel human experimenter, they subsequently approached that experimenter faster than when the screen had been turned away from the experimenter, who looked at a clipboard instead. Moreover, when pairs of chimpanzees watched a video together, they spent more time in the same part of their enclosure afterward than when they had been watching a similar video individually on separate screens. These results demonstrate that at least some of the psychological underpinnings of people’s capacity to bond through shared experiences are also present in other animals.

There seems to be, however, a subtle yet potentially important difference between how humans bond through shared experiences and how this works for other animals. In the studies on shared experiences and social bonding discussed so far, participants (ape and human) had all the information necessary to infer that their partner’s experience was psychologically similar to their own (i.e., “We experience X”). As mentioned earlier, however, human sharing often seems to involve some mutualistic awareness of their experience being shared, creating a shared representation (i.e., “I know we both know we are attending to X”) by mutually affirming that this is indeed the case (e.g., linguistically or through communicative eye contact; see Siposova et al., 2018). One plausible hypothesis is, therefore, that humans have a unique capacity to bond with others by creating such mutualistic shared (metacognitive) representations during (and about) their social activities.

Recent comparative research tested this hypothesis (Wolf & Tomasello, 2020a): Human children and great apes (chimpanzees and bonobos) engaged in an experiment in which they watched something with an experimenter. However, in the experimental condition, the experimenter made communicative eye contact (see Siposova et al., 2018) with the participants, providing participants with the necessary information to infer that both they and their partner knew that they both realized they were watching the video together, allowing participants to create a mutualistic shared metacognitive representation about their experience being shared. In the control condition, however, there was no communicative eye contact in response to the video starting (experimenters made eye contact at a later point in the procedure, when they reconvened in the same room, to control for the effect of eye contact on social bonding in general). In the control condition, participants thus merely observed their partner watching the same video, allowing them to infer that their psychological experience was similar to that of their partner (i.e., the participant knew they were both watching the video). However, they did not have the information necessary to infer that their partner was aware of this also, making it unlikely they created a mutualistic shared representation about watching the video (i.e., participants did not know whether their partner knew they were watching the video together). Subsequent approach-based social-bonding measures that had previously been shown to work for both children and great apes (Wolf & Tomasello, 2019, 2020b) demonstrated that human children, but not great apes, were more willing to interact with a partner with whom they had created common ground about their experience being shared (Wolf & Tomasello, 2020a).

These findings suggest that humans indeed have a unique capacity to create social closeness with others by creating mutual awareness of their experience being shared. Thus, these results align with a broader shared-intentionality framework in which the consequences of humans’ sophisticated capacity to understand and relate to others has important consequences for human social behavior not only in terms of cooperation, communication, and social learning but also for the way in which humans create social bonds with others.

## The Psychology of Bonding Through Shared Intentionality

So far, we have provided an explanation for the evolution of the unique aspects of human social bonding by (a) placing the emergence of this aspect of human sociality in the broader context of human social-cognitive evolution and (b) proposing that the specific social-cognitive mechanisms that emerged during this part of human social-cognitive evolution and shape many of humans’ unique forms of communication and cooperation also fundamentally shape the way in which humans bond with others. Thus, the crucial questions that remain pertain to why and how these cognitive mechanisms cause social bonding during these unique social activities to occur and why this way of social bonding acquired such a prominent place in people’s social lives over the course of human social evolution.

One simple potential explanation for why so many of people’s social-bonding activities rely on shared experiences is that because engaging in collaborative interactions is so important to the human species, humans created a proximal reward mechanism that causes them to experience sharing intentional states during such interactions as rewarding. Given that even young children already prefer to act together over acting alone (Rekers et al., 2011) regardless of their outcomes, there might be some truth to this. Moreover, the formation of shared representations might give people more certainty about the world around them (e.g., perceiving others’ responses to things in the world to be similar to one’s responses allows one to be more certain one is responding to them the right way). This increased epistemic confidence and sense of control about the world around people might also cause them to experience sharing representations as more rewarding and evaluate these interactions and their interaction partner more positively (Higgins et al., 2021). However, although experiencing shared representations as psychologically rewarding would make humans more likely to engage in social activities in general, it does not explain why humans choose to act with the same individuals over and over again and prefer interacting with these individuals over others, which constitutes the essence of a social bond.

Another, more low-level cognitive explanation is that the shared representations formed during shared experiences might decrease the cognitive demands of understanding the reasons behind a partner’s behavior and predicting the partner’s behavior in the future because one can simply assume that the mental states underlying that behavior are similar to one’s own. Because the ease with which information is processed (i.e., processing fluency) has long been shown to cause individuals’ attitudes toward that information and things associated with this information to become more positive (Alter & Oppenheimer, 2006, 2009; Bornstein, 1989; Gordon & Holyoak, 1983; Laham et al., 2009; Reber et al., 2004; Reber & Unkelbach, 2010; Song & Schwarz, 2009; Wilson & Miller, 1961; Wilson & Nakajo, 1965; Zajonc, 1968), this idea might simply also apply to interaction partners: People with whom one shares mental states and creates shared representations are easier to interact with, which causes one to evaluate them more positively and anticipate more rewarding future social interactions (i.e., providing the basis of a social bond). Yet this is also unlikely to be the full story because simple representations of coexperience (e.g., “We feel X”) should be sufficient for this to work. Yet research suggests that human social bonding seems to be specifically effective because of the creation of mutualistic shared representations during social interactions/activities. So why is this mutualistic metacognitive sharing of experience, then, so effective in creating social bonding?

A plausible answer to this question is that shared experience-based social activities provide people with valuable information about (potential) collaborative partners or group members. One important function of establishing social relationships is acquiring stable access to social support and potential collaborative partnerships (Seyfarth & Cheney, 2012). The effectiveness of such a partnership, at least for human collaboration, relies heavily on people’s ability to share mental states during communication (e.g., understand the shared meaning of one’s communicative acts) and collaborative decision-making (e.g., inferring a shared goal both individuals are willing to commit to). This ability might vary substantially between partners so that one needs to select partners carefully. Social activities built around shared experiences might thus provide a relatively low-stakes testing ground for potential partners’ collaborative prowess, which might look like, “Is it easy to understand and predict my partner’s behavior, and does my partner understand why we are doing what we are doing? Is my partner sufficiently communicative about what they want and how they want to do it?” By engaging in relatively low-stakes social activities with a variety of potential social partners, people can evaluate which individuals are most predictable and communicative and thus suit their collaborative needs best.

In addition, engaging in these social activities might also tell people something about how much others are invested in them as potential social partners. Creating shared representations (e.g., “I know we know we feel good about this game”) during interactions requires a mutual acknowledgment of the experience being shared. This social act is, in principle, cooperative and also socially selective (i.e., aimed at a specific individual). Thus, when an individual attempts to share an experience with a partner in this way, this is usually a genuine expression of social interest, affiliative motivation, and nonaggressive intentions toward this particular (potential) partner, providing a solid psychological basis for subsequent bonding to occur. Moreover, if individuals spend time and energy on creating shared social activities that could also be done alone (e.g., watching a movie), then this is, in essence, a form of costly signaling: Spending time and resources to create and partake in such shared experiences shows social partners that one is invested in sharing this experience specifically with them and thus that they are perceived as valuable social partners. As such, these types of interactions have essentially become a triadic form of social grooming unique to humans.

## Implications

Humans’ capacity to create sophisticated shared representations thus expanded the ways in which they are able to bond with others. First, it transformed the social-bonding mechanism humans share with other animals, for example, by causing communicative eye contact and cooperative communication to become more important when bonding with others by seeking out their proximity, physical contact, or sexual or playful interactions. Second, the emergence of people’s capacity to bond with others by sharing experiences with them in common ground also motivated humans to engage in novel social activities specifically suited to share experiences with others, for example, by seeking out or creating interesting external stimuli to experience together (e.g., music, stories, or rituals) or using linguistic communication to share mental content with each other (O’Madagain & Tomasello, 2019) during personal conversations or gossip. It also allowed humans to create the representations necessary for cooperatively structured competitive play in which the rules that structure this play are shared and agreed upon, for example in a football game: “We are both aware that we both know the rule that if the ball goes into the goal of one team, the other team gets a point.” Because if this is not known to be known to both parties, no goal would ever be scored.

Thus, this idea not only helps researchers better understand why humans engage in such peculiar social-bonding activities, but it also brings the field one step closer to bridging the bonding gap in terms of social-network size (Dunbar, 2012). The uniquely human capacity to create shared representations of their experiences enables humans to bond more efficiently than other primates because creating shared representations can be done (and often inadvertently occurs) during collaborative activities that are crucial for human survival (e.g., food gathering), whereas other primates that rely mostly on physical interactions such as grooming for social bonding need to more carefully allocate their time between activities that facilitate social bonding and those directly necessary for survival.

Shared experiences also provide a relatively safe way to bond with others, reducing the risk of potential negative consequences (e.g., aggression, pathogen transmission) inherent to more physical forms of social bonding such as social grooming. Thus, where this poses a serious risk for species in which physical interaction is the primary component of social bonding, such as other primates (Lehmann et al., 2007), this is no longer the case for humans. Instead, humans’ unique social-cognitive capacities allow them to reserve social activities that entail high levels of physical touch predominantly for individuals with whom they have stronger, more intimate social bonds (Suvilehto et al., 2015).

Within these stronger social bonds, physical touch itself then also becomes a potential source of information to infer triadic engagement and create shared representations. For example, although in Western societies triadic engagement, especially during child rearing, mostly occurs face to face and includes individuals alternating between a sustaining a shared focus on an object and making eye contact (Mundy, 2006), in other cultures, such as the Vanuatu in Melanesia, research has shown that triadic engagement, at least with children, happens relatively more through touch (Little et al., 2016). Thus, there may be substantial cultural variability in how the information required to create shared representations is transferred between individuals, potentially resulting in highly variable manifestations of social-bonding practices and activities across cultures in terms of, for example, eye contact, touch, or the use of language.

More generally, this example raises an important issue related to empirical research into this part of human psychology. Researchers studying social bonding through shared representations need to be mindful about which information they provide to their participants and what inferences participants can draw from them. Is the information about which a mental state can be shared publicly available? Are participants supposed to infer from instructions that others are sharing an experience with them and will they, in these cases, only know that others are going through the same experience? Or will they be told that the other participants know this as well? And if the information is publicly available, can participants make eye contact? Are they allowed to communicate? In other words, will the design of the procedure allow participants to infer that they both know that they are experiencing the same thing? Or are they merely inferring that others are going through a similar experience? These issues are key to decide on if one wants to disentangle the psychological and behavioral effects of simply coexperiencing something versus creating mutualistic metacognitive shared representations with others.

Overall, the current account highlights the importance of humans’ unique social-cognitive skills in people’s social and mental well-being. Human capacity to create shared intentionality with others has long been argued to be important in people’s social lives, specifically within social development. However, until now, this has been proposed mostly in the context of social competencies in the realm of communicative development (Baldwin, 1995; Tomasello, 2008) and cooperation (e.g., compliance, empathy, or prosocial peer interactions; Vaughan Van Hecke et al., 2007). This current account extends this view by proposing that the development of children’s capacity to engage in shared intentionality might also be directly related to their socio-emotional development and potentially predictive of their social behavior. Early variability in the capacity to share mental states, such as joint attention, might predict social-network size, loneliness, and relationship quality later in life, posing an interesting direction for future research.

The effect of individual differences in the capacity to share mental states on social outcomes seems particularly relevant for clinical populations in which the development of these skills is often impaired, such as children with autism spectrum disorder (ASD; Mundy, 2006; Mundy & Newell, 2007). Research has shown that children with ASD experience more loneliness and have lower-quality friendships that neurotypical children (Bauminger et al., 2003; Bauminger & Kasari, 2000). Perhaps children with ASD might have more difficulty creating shared representations, making it harder for them to initiate, recognize, and respond to shared experiences, for example, through joint attention. This, in turn, might make it more difficult for them to bond with others in these ways. Thus, better understanding the social cognition underlying this part of human social-bonding behavior might contribute to the development of interventions for children with ASD aimed at helping them understand and recognize the difference between when they experience something, when they experience it together, and when both they and their partner know they are experiencing something together.

## Conclusion

Social bonding is one of the most fundamental aspects of human psychology. Several theoretical accounts of this psychology (i.e., attachment and belonging theory) have been immensely valuable for understanding human social bonding as part of a phylogenetic continuum in which people share much of this psychology with other primates through common descent. Yet none of these accounts provide a psychological explanation for why a substantial part of humans’ social-bonding activities is so different from that of humans’ nonhuman primate cousins. The current shared intentionality account of humans’ unique social bonding provides this explanation by placing the evolution of human social bonding in the larger context of humans’ social-cognitive evolution. The sophisticated social-cognitive capacities to create shared representations necessary for complex forms of communication (e.g., language) and cooperation also changed the way in which people form and maintain relationships with others. Specifically, this psychology allows people to engage in triadic social grooming through social activities built around mutualistic metacognitive shared experiences, providing important information about who might be capable social partners and which of these partners would be interested in providing and/or reciprocating social support in the future. Understanding and studying human social bonding from this perspective might thus help to better understand the unique complexities of human social life.

## Transparency

*Action Editor:* Leonel Garcia-Marques

*Editor:* Interim Editorial Panel
